# Activation of Mesenchymal Stem Cells by Macrophages Prompts Human Gastric Cancer Growth through NF-κB Pathway

**DOI:** 10.1371/journal.pone.0097569

**Published:** 2014-05-13

**Authors:** Tingting Yang, Xu Zhang, Mei Wang, Jie Zhang, Feng Huang, Jie Cai, Qiang Zhang, Fei Mao, Wei Zhu, Hui Qian, Wenrong Xu

**Affiliations:** 1 School of Medical Science and Laboratory Medicine, Jiangsu University Zhenjiang, Jiangsu, China; 2 The Affiliated Hospital of Jiangsu University, Zhenjiang, Jiangsu, China; Sapporo Medical University, Japan

## Abstract

Accumulating evidence indicate that macrophages activate mesenchymal stem cells (MSCs) to acquire pro-inflammatory phenotype. However, the role of MSCs activated by macrophages in gastric cancer remains largely unknown. In this study, we found that MSCs were activated by macrophages to produce increased levels of inflammatory cytokines. Cell colony formation and transwell migration assays revealed that supernatants from the activated MSCs could promote both gastric epithelial cell and gastric cancer cell proliferation and migration. In addition, the expression of epithelial-mesenchymal transition (EMT), angiogenesis, and stemness-related genes was increased in activated MSCs. The phosphorylated forms of NF-κB, ERK and STAT3 in gastric cells were increased by active MSCs. Inhibition of NF-κB activation by PDTC blocked the effect of activated MSCs on gastric cancer cells. Co-injection of activated MSCs with gastric cancer cells could accelerate gastric cancer growth. Moreover, human peripheral blood monocytes derived macrophages also activated MSCs to prompt gastric cancer cell proliferation and migration. Taken together, our findings suggest that MSCs activated by macrophage acquire pro-inflammatory phenotype and prompt gastric cancer growth in an NF-κB-dependent manner, which provides new evidence for the modulation of MSCs by tumor microenvironment and further insight to the role of stromal cells in gastric carcinogenesis and cancer progression.

## Introduction

Gastric cancer is one of the most frequently occurring malignancies and keeps a major cause of cancer mortality all over the world [1], [2]. In China, there are about 360,000 individuals die of gastric cancer every year [3]. Though the incidence has decreased in recent years in the West, the survival is still worse [4]. Over the past decades, great effort has been exerted to elucidate the pathogenesis of gastric cancer. However, the complex mechanism of gastric carcinogenesis is still uncovered. Accumulating evidence indicate that long-term chronic inflammation is one of the leading causes of tumorigenesis. Release of pro-inflammatory mediators and increased local levels of oxygen and nitrogen species can contribute to carcinogenesis [5]. The dysregulated production of cytokines in inflammatory microenvironment stimulates the expression of genes associated with cancer development and modifies structural features of microenvironment to accelerate cancer initiation and progression [6]–[9].

Tumor microenvironment consists of various stromal cells, including infiltrating immune cells, carcinoma-associated fibroblasts (CAFs), mesenchymal stem cells (MSCs), and blood and lymphatic vascular networks. These cells interact with each other and constitute inflammatory microenvironment and contribute to tumorigenesis [10], [11]. Among the stromal cells, macrophages, as important immune regulatory cells, play a dominant role in managing inflammation in tumor microenvironment. For example, macrophages isolated from tumor microenvironment of breast cancer patients secret chemotactic cytokines to augment metastasis of carcinoma cells [12]. Macrophages have also been shown to promote inflammatory response and tumorigenesis through impacting on expression of inflammatory cytokines and altering the molecular oncogenic programs within epithelial cells [13].

Mesenchymal stem cells (MSCs) are another major component of the tumor microenvironment and are considered as the precursor cells of cancer associated mesenchymal cells and endothelial cells [14]. The previous studies have indicated that MSCs secret soluble factors to promote cancer cell proliferation and metastasis [10]. In an inflammation-associated gastric cancer model, MSCs could be activated towards CAFs to increase chronic inflammation and cancer progression [15]. Furthermore, MSCs have been reported to recruit monocytes/macrophages to promote tumor growth in a CCR2-depedent manner [16]. Interactions between macrophages and MSCs produce an activated, pro-inflammatory phenotype with high CXCL10 and IL-6 secretion, which may influence the inflammatory microenvironment [17].

Gastric cancer is a classic model of chronic inflammation to cancer. However, the role of MSCs activated by macrophage in gastric cancer and the underlying mechanism are still largely unknown. In this study, we found that MSCs were strongly activated by macrophages under inflammatory condition, to produce inflammatory cytokines and tumor-promoting factors, leading to the enhancement of gastric epithelial cell and cancer cell proliferation and migration through the activation of NF-κB pathway. Our results indicate that macrophages-activated MSCs promote gastric cancer growth and progression under inflammatory condition.

## Materials and Methods

### Cell Culture

Human gastric cancer cell line HGC-27, human gastric epithelial cell line GES-1, and human acute monocytic leukemia cell line THP-1 were purchased from the Institute of Biochemistry and Cell Biology at the Chinese Academy of Sciences (Shanghai, China). GES-1 and THP-1 cells were cultured in RPMI-1640 medium (Invitrogen, Carlsbad, CA, USA) with 10% fetal bovine serum (FBS, Invitrogen), and HGC-27 cells were maintained in high-glucose DMEM (H-DMEM, Invitrogen) with 10% FBS. MSCs were derived from umbilical cord and cultured in low-glucose DMEM (L-DMEM, Invitrogen) with 10% FBS. Cells were all incubated at 37°C in humidified cell culture incubator with 5% CO_2_. The fresh umbilical cord tissues were collected from healthy puerperal mothers after written informed consent was obtained. MSCs were isolated and characterized as previously described [18]. All experiment protocols were approved by the Ethics Committee of Jiangsu University. MSCs at passage 3 were selected for the experiments.

### Supernatant Preparation

For the preparation of macrophage associated MSCs supernatant, MSCs (3×10^5^) were seeded to adhere. THP-1 cells (1∶1 ratio) were added with fresh medium in the presence or absence of LPS (1 µg/ml). After co-culture for 48 h, the medium was discarded and the cells were gently washed with PBS to remove THP-1 cells. The supernatants from activated MSCs were collected 24 hours later, filtered with a 0.22-um filter and stored at −80°C until use. When used for cell function analyses, the supernatants from activated MSCs were mixed with equal volume of 10% FBS-containing H-DMEM or RPMI-1640 medium. For signaling pathway analyses, cells were treated with the supernatants from activated MSCs for 1 h.

### Luminex Assay

Human cytokine & chemokine magnetic bead panel kit (#HCYTOMAG-60K) (Millipore, Billerica, MA, USA) was designed to detect granulocyte colony stimulating factor (G-CSF), platelet derived growth factor-BB (PDGF-BB), monocyte chemoattractant protein-1 (MCP-1), tumor necrosis factor-α (TNF-α), vascular endothelial growth factor (VEGF), IL-10, IL-1β, IL-4, IL-6 and IL-8 in the supernatant from activated MSCs. All procedures were processed according to the manufacturer’s instruction. The signals were detected and analyzed by using Luminex200 System (Millipore).

### Transwell Migration Assay

After treatment with the supernatants from activated MSCs for 48 h, GES-1 and HGC-27 cells (1×10^5/^well) were put into the upper chamber (8 µm) (Corning, NY, USA) in serum-free medium. The complete medium was placed into the lower chamber. After incubation for 12 h, cells remaining at the bottom of the polycarbonate membrane were wiped off with cotton swabs. The cells migrating to the lower surface of the membrane were then fixed with methanol for 30 min. The migrated cells were stained with crystal violet for 15 min and counted in six random fields under the microscope (100×).

### Cell Colony Formation Assay

Cells were collected after treatment with the supernatants from MSCs for 48 h and seeded into 6-well plates (1000 cells/well) and cultured for 10 days. The medium was changed every three days. At the end of the growth period, cells were fixed with methanol for 30 min and stained with crystal violet for 15 min. The cell colonies were photographed and the number of colonies was counted for statistical analysis.

### RNA Extraction and Real-time PCR

Total RNA was extracted using Trizol reagent (Invitrogen) according to the manufacturer’s instructions. Five microgram of RNA was used for quantitative real-time PCR analysis. β-actin was used as an internal control. The sequences of specific primers were all listed in Table S1.

### Western Blot

Cells were lysed and homogenized in RIPA buffer supplemented with complete protease inhibitors. Equal amount of proteins (200 µg) was resolved in 12% SDS-PAGE. The proteins were then transferred onto PVDF membranes following electrophoresis. After blocked in 5% (w/v) non-fat milk for 1 h at room temperature, the membranes were then incubated at their respective appropriate dilutions of specific primary antibodies overnight at 4°C. The sources of primary antibodies were: anti-Oct4, anti-Sox2, anti-Vimentin and anti-phospho-STAT3 (Signalway Antibody, USA), anti-GAPDH (Kangcheng, Shanghai, China), anti-E-cadherin, anti-ERK1/2, anti- phospho-ERK1/2 and anti-N-cadherin (Santa Cruz Biotechnology, Santa Cruz, CA, USA), anti-PCNA, anti-STAT3, anti-NF-κB, anti-phospho-NF-κB, anti-CyclinD1, anti-VEGF and anti-C-Jun (Bioworld Technology, Louis Park, MN, USA), anti-C-myc (ProteinTech Group, Chicago, IL, USA).

### Animal Model

Three to five-week-old BALB/c nude mice were purchased from Slac Laboratory Animal Center (Shanghai, China). Animals were maintained in accordance with institutional policies, and all studies were performed with approval of the University Committee on Use and Care of Animals of Jiangsu University. HGC-27 cells (1×10^6^) and MSCs (5×10^5^) were trypsinized, washed and resuspended in 200 µl PBS, respectively. Then the cells were mixed and co-injected into the left flank of nude mice. Tumors were surgically removed 20 days after injection, photographed and weighted. Tumor size was assessed by caliper measurement and calculated based on the modified ellipsoid formula (L×W×W/2), where L represents length, and W represents width.

### Immunohistochemistry

Formalin-fixed paraffin-embedded mouse tumor tissue sections were first deparaffinized in xylene, rehydrated through graded ethanol. The sections were boiled for 10 min in citrate buffer (pH 6.0, 10 mM) for antigen retrieval. The endogenous peroxidase activity was inhibited with exposure to 3% hydrogen peroxide for 10 min. Then the sections were blocked with 5% BSA (Boster Bioengineering, Wuhan, China) and incubated with proper diluted PCNA or VEGF primary antibody at 37°C for 1 h. After washed with PBS, the sections were then incubated with diluted secondary antibody for 20 min. Finally, sections were visualized with 3,3′-diaminobenzidine (DAB) and then counterstained with hematoxylin for examination by light microscopy (200×).

### Primary Human Monocytes Isolation

Human monocytes were obtained from buffy coat of peripheral blood samples donated by healthy donor using Ficoll (Histopaque-1077) (Sigma, USA). Fresh RPMI 1640 supplemented with 10% FBS were changed every 2 days, and non-adherent cells were removed and purified. Monocytes were incubated for 7 days and 50 ng/ml M-CSF was added to obtain macrophages (M). Then 24 h supernatant secreted by macrophages was collected and filtered. Adherent MSCs were treated with the macrophage supernatant in the absence or presence of LPS (1 µg/ml) for 48 h and washed. The supernatants from activated MSCs were collected 24 h later and used for following studies.

### Statistical Analysis

Statistical analysis was done with SPSS Statistics software 16.0. Data were presented as mean ± SD. Differences in different groups were analyzed using one-way ANOVA. Differences between PDTC treatments were tested by *t* test. Statistical *P* value<0.05 was considered to be significant.

## Results

### Co-culture with Macrophages Under Inflammatory Condition Up-regulated the Expression of Inflammatory Cytokine and Stemness Genes in MSCs

**Figure 1:**
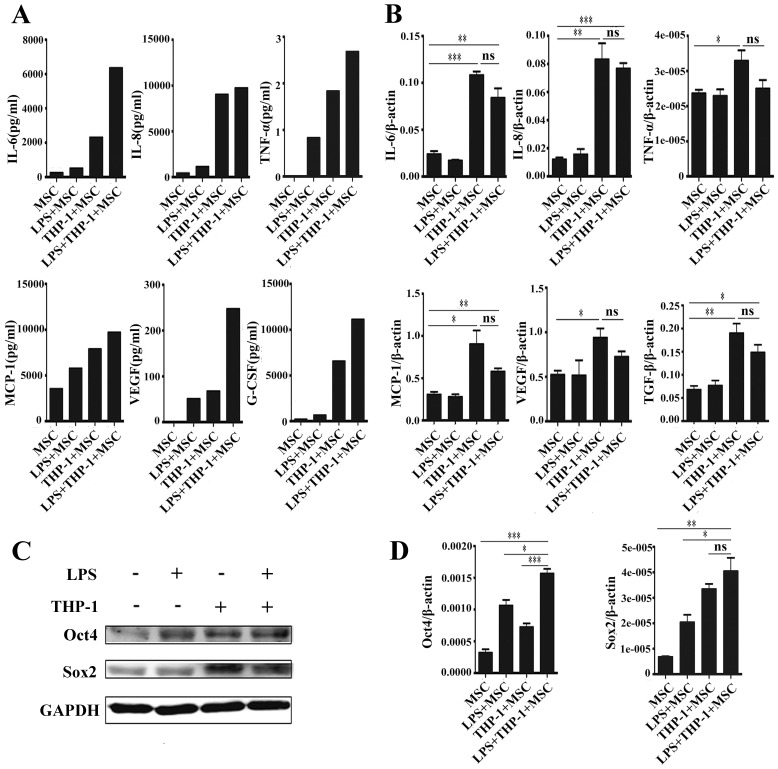
The expression of inflammatory cytokines and stemness genes in MSCs were up-regulated after co-culture with macrophages and LPS stimulation. (A) Luminex assay for IL-6, IL-8, TNFα, MCP-1, VEGF and G-CSF levels in the supernatants from MSCs. (B) Real-time PCR analyses of IL-6, IL-8, TNFα, MCP-1, VEGF and TGF-β mRNA expression in MSCs. ***P<0.001, **P<0.01, *P<0.05. (C) Western blot analyses of Oct4 and Sox2 protein levels in MSCs. (D) Real-time PCR analyses of Oct4 and Sox2 mRNA expression in MSCs. ***P<0.001, **P<0.01, *P<0.05.

To investigate the effect of macrophages on MSCs under inflammatory condition, we co-cultured MSCs with THP-1 cells in the absence or presence of LPS (1 µg/ml) for 48 h. THP-1 cells were removed by PBS washing and the adherent MSCs were cultured in fresh medium for additional 24 h (Figure S1). Luminex assay was conducted to determine the levels of several inflammatory factors in the supernatants from MSCs. The results showed that the production of IL-6, IL-8, TNF-α, MCP-1, VEGF and G-CSF was significantly increased in the supernatant from MSCs co-cultured with THP-1 cells in the presence of LPS. Low or undetectable levels of these cytokines were observed in MSCs that were not co-cultured with THP-1 cells (Figure 1A). To confirm the increased expression of these inflammatory cytokines, we preformed real-time PCR to detect the mRNA levels of these cytokines. We found that in consistent with the Luminex assay results (Figure 1B), co-culture with THP-1 cells up-regulated the mRNA levels of IL-6, IL-8, and TNF-α in MSCs. To determine whether co-culture with THP-1 cells affects the stemness of MSCs, we detected the expression of Oct4 and Sox2 in MSCs by using Western blot. The results showed that both Oct4 and Sox2 protein levels were increased in MSCs that were co-cultured with THP-1 cells (Figure 1C). To further confirm this phenomenon, we also examined the mRNA levels of Oct4 and Sox2 in MSCs. The results of real-time PCR showed that co-culture with THP-1 cells up-regulated the expression of Oct4 and Sox2 genes in MSCs (Figure 1D). In general, these results suggest that MSCs were significantly activated to produce higher levels of inflammatory cytokines by co-cultured THP-1 cells under inflammatory condition.

### Macrophages-activated MSCs Enhanced the Proliferation and Migration of Gastric Epithelial Cells

**Figure 2:**
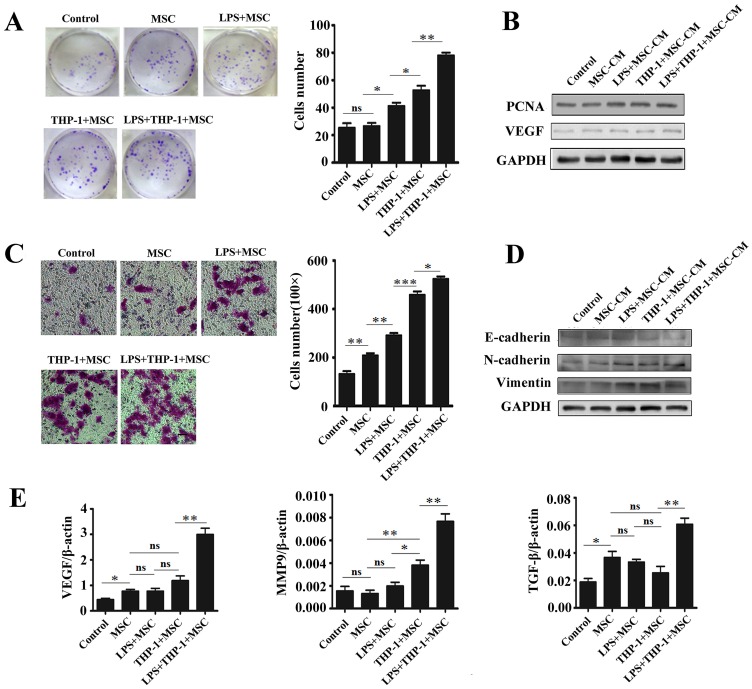
Macrophages-activated MSCs under inflammatory condition promoted the proliferation and migration of GES-1 cells. (A) Representative images of cell colony formation assay and histogram of colony number. Data were listed as mean±SD of three wells. **P<0.01, *P<0.05. (B) Western blot assays for PCNA and VEGF protein levels. (C) Representative images of transwell migration assay and histogram of the number of migrated GES-1 cells. Magnification, ×100, scale bar = 50 µm. ***P<0.001, **P<0.01, *P<0.05. (D) Western blot assays for E-cadherin, N-cadherin and Vimentin protein levels. (E) Real-time PCR analyses of VEGF, MMP9 and TGF-β mRNA expression. **P<0.01, *P<0.05.

Macrophages and MSCs are important components of tumor microenvironment. To demonstrate the functional roles of macrophages-activated MSCs, we collected the supernatants from activated MSCs and incubated with gastric epithelial cell line GES-1 for 48 h. We then performed cell colony formation assay to evaluate the proliferation of GES-1 cells. As shown in Figure 2A, the cells incubated with the supernatant from activated MSCs grew faster and formed more and larger colonies than those in other groups. In addition, incubation with the supernatant from activated MSCs also increased the protein levels of PCNA and VEGF in GES-1 cells (Figure 2B). The results of transwell migration assay showed that the number of migrated GES-1 cells in activated MSCs plus LPS group was more than that in other groups (Figure 2C). The results of Western blot analyses showed that macrophages-activated MSCs increased the expression of mesenchymal cell markers (N-cadherin and Vimentin) and decreased the expression of epithelial cell marker (E-cadherin) in GES-1 cells (Figure 2D). We next determined the expression of VEGF, MMP9 and TGF-β by using real-time PCR. As shown in Figure 2E, incubation with the supernatant from activate MSCs plus LPS group up-regulated the expression of VEGF, MMP9 and TGF-β in GES-1 cells. Thus, MSCs activated by macrophages in inflammatory environment significantly promoted gastric epithelia cell proliferation and migration.

### Macrophages-activated MSCs Promoted the Growth and Migration of Gastric Cancer Cells

**Figure 3:**
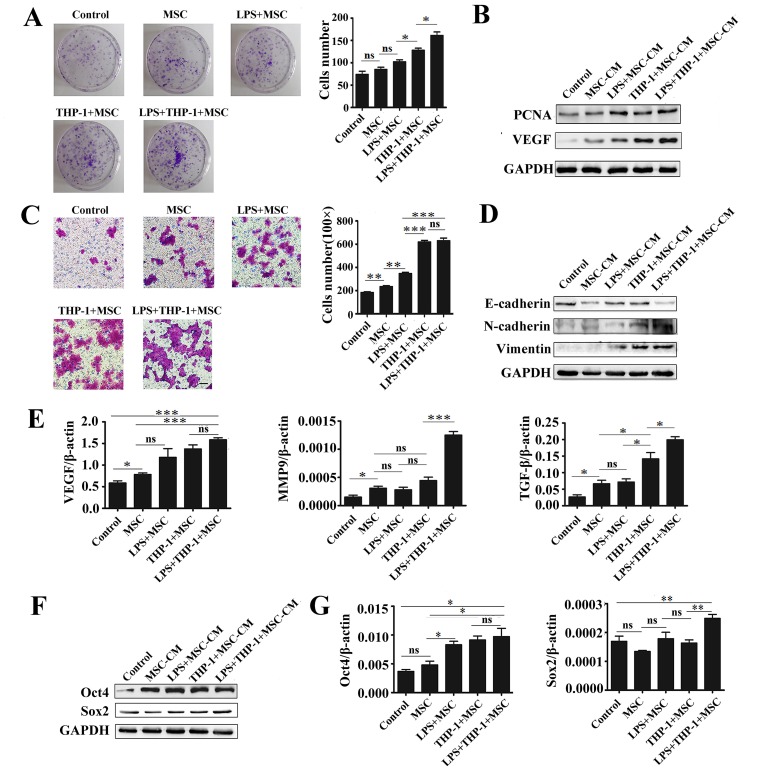
Macrophages-activated MSCs under inflammatory condition promoted the proliferation and migration of HGC-27 cells. (A) Representative images of cell colony formation assay and histogram of colony number. Data were listed as mean±SD of three wells. *P<0.05. (B) Western blot assays for PCNA and VEGF protein levels. (C) Representative images of transwell migration assay and histogram of the number of migrated HGC-27 cells. Magnification, ×100; scale bar = 50 µm. ***P<0.001, **P<0.01. (D) Western blot assays for E-cadherin, N-cadherin and Vimentin protein levels. (E) Real-time PCR analyses of VEGF, MMP9 and TGF-β mRNA expression. ***P<0.001, *P<0.05. (F) Western blot assay for Oct4 and Sox2 protein levels in HGC-27 cells. (G) Real-time PCR analyses of Oct4 and Sox2 mRNA expression. **P<0.01, *P<0.05.

Since macrophages-activated MSCs affect gastric epithelial cell proliferation and migration, we next determined the effect of macrophages-activated MSCs on human gastric cancer HGC-27 cells. The results of cell colony formation assay showed that HGC-27 cells incubated with the supernatant from macrophages-activated MSCs grew faster and formed larger clones than the other groups (Figure 3A). Western bolt analyses showed that the protein levels of PCNA and VEGF in HGC-27 cells were significantly up-regulated by the supernatant from macrophages-activated MSCs (Figure 3B). Transwell migration assay revealed that the number of migrated HGC-27 cells was strikingly increased by the supernatant from macrophages-activated MSCs (Figure 3C). Western blot analyses showed that macrophages-activated MSCs induced the expression of mesenchymal cell markers (N-cadherin and Vimentin) and inhibited the expression of epithelial cell marker (E-cadherin) in HGC-27 cells (Figure 3D). The mRNA levels of VEGF, MMP9 and TGF-β were also increased in HGC-27 cells after treatment with the supernatant from macrophages-activated MSCs (Figure 3E). Considering that cancer cell stemness is strongly linked to their migration, we detected the expression of stemness genes in HGC-27 cells. The expression of Oct4 and Sox2 was notably up-regulated by the supernatant from macrophages-activated MSCs (Figure 3F). In consistent with the real-time PCR results, the protein levels of Oct4 and Sox2 were also increased in HGC-27 cells by the supernatant from macrophages-activated MSCs (Figure 3G). In brief, these results suggest that macrophages-activated MSCs also promoted gastric cancer cell growth, migration through the induction of EMT and cell stemness under inflammatory condition.

### Macrophages-activated MSCs Promoted Gastric Cell Proliferation and Migration Through NF-κB Pathway

**Figure 4:**
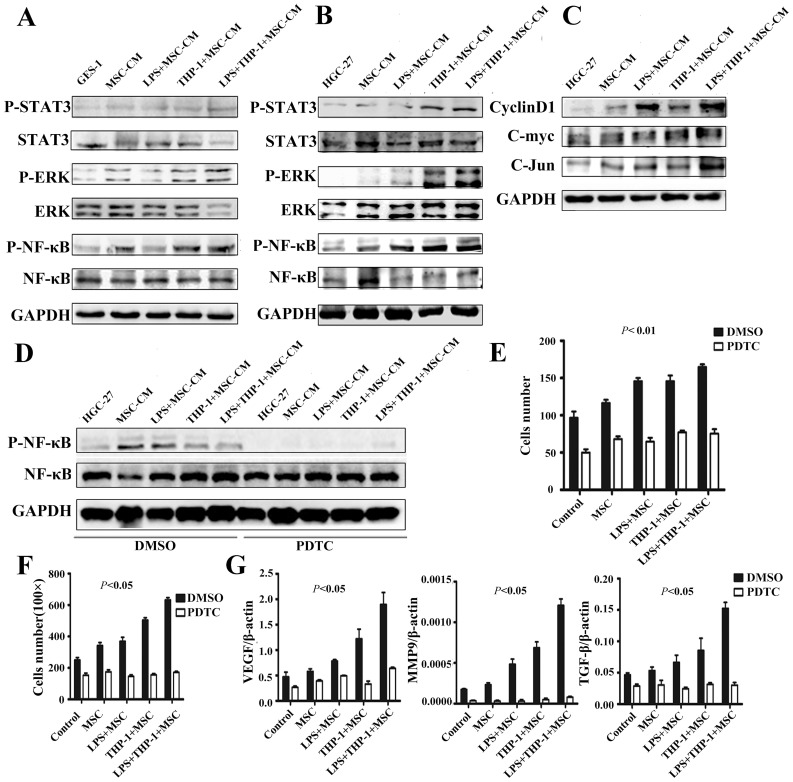
Macrophages-activated MSCs enhanced cell proliferation and migration through NF-κB activation. (A) GES-1 and (B) HGC-27 cells treated with the supernatants for activated MSCs. The expression of p-STAT3, STAT3, p-ERK, ERK, p-NF-κB, and NF-κB proteins were determined by using Western blot. (C) Western blot assays for Cyclin D1, C-myc, and C-Jun protein levels in HGC-27 cells. (D) Western blot assays for p-NF-κB and NF-κB protein levels in HGC-27 cells that were treated with the supernatants from the activated MSCs in the presence or absence of PDTC (100 nM). The number of cell colonies (E) and migrated cells (F) for HGC-27 cells treated with different MSCs supernatants in the presence or absence of PDTC (100 nM). (G) Real-time PCR analyses of VEGF, MMP9, and TGF-β mRNA expression in HGC-27 cells treated with different MSCs supernatants in the presence or absence of PDTC (100 nM). **P<0.01, *P<0.05.

In order to explore the mechanism responsible for the promoting role of macrophages-activated MSCs in cell proliferation and migration, we determined the expression of several key signaling transducers for inflammation and cancer, including STAT3, ERK and NF-κB, in gastric epithelial cells and gastric cancer cells. The results of Western blot analyses showed that the levels of p-STAT3, p-ERK and p-NF-κB were significantly higher in GES-1 cells treated with the supernatants from activated MSCs than those in other groups. The protein level of NF-κB had no change while that of ERK and STAT3 slightly decreased (Figure 4A). The increases in p-STAT3, p-ERK and p-NF-κB protein levels were also observed in HGC-27 cells after treatment with the supernatants from the activated MSCs (Figure 4B). We confirmed the up-regulation of p-STAT3, p-ERK and p-NF-κB protein levels by the supernatants from the activated MSCs in another gastric cancer cell line SGC7901 (Figure S2). To determine whether the downstream target genes were also activated in HGC-27 cells, we examined the expression of Cyclin D1, C-myc and C-Jun proteins. As shown in Figure 4C, the protein levels of Cyclin D1 and C-Jun were elevated after treatment with the supernatants from the activated MSCs.

To future determine whether NF-κB plays a major role in the functions of activated MSCs, HGC-27 cells were pre-treated with PDTC to inhibit NF-κB phosphorylation and then incubated with the supernatants from the activated MSCs. We found that the induction of NF-κB phosphorylation by activated MSCs in HGC-27 cells was markedly inhibited by PDTC (Figure 4D). The results of cell colony formation and transwell migration assays showed that the enhanced proliferation and migration of HGC-27 cells by the activated MSCs were significantly reduced in PDTC-treated groups (Figures 4E and 4F). Furthermore, PDTC treatment also inhibited the up-regulation of VEGF, MMP9 and TGF-β by activated MSCs in HGC-27 cells (Figure 4G). Taken together, these results indicate that the activated MSCs prompt gastric epithelial cell and gastric cancer cell proliferation and migration through NF-κB.

### Macrophages-activated MSCs Promoted Gastric Cancer Growth in vivo

**Figure 5:**
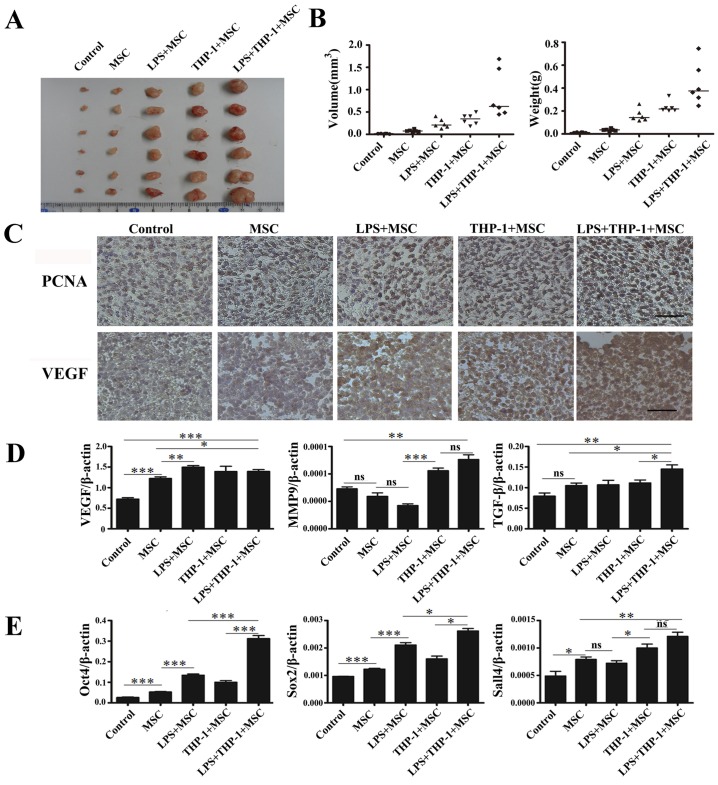
Macrophages-activated MSCs promoted gastric cancer growth *in vivo*. (A) Representative images of the xenograft tumor tissues and (B) the volume and weight of tumor tissues removed from mice injected with HGC-27 cells alone or together with MSCs. (C) Immunohistochemical analyses of PCNA and VEGF protein levels in tumor tissues. Magnification, ×200; scale bar = 50 µm. (D) Real-time PCR analyses of VEGF, MMP9, and TGF-β mRNA expression in tumor tissues. ***P<0.001, **P<0.01, *P<0.05. (E) Real-time PCR analyses of Oct4, Sox2, and Sall4 mRNA expression in tumor tissues. ***P<0.001, **P<0.01, *P<0.05.

Given the evidence that macrophages-activated MSCs could enhance gastric cell proliferation *in vitro*, we wondered whether these MSCs exerted promoting role in gastric cancer growth *in vivo*. Thus, we co-injected HGC-27 cells with the activated MSCs into nude mice. Twenty days later, tumor tissues were removed, measured and weighted. As shown in Figures 5A and 5B, the xenograft tumors in activated MSCs co-injected group were larger than those in other groups. Immunohistochemical analyses were performed to determine the expression of growth-related proteins and pro-angiogenic factors in tumor tissues. The stronger positive staining of PCNA and VEGF was observed in activated MSCs co-injected group (Figure 5C). Real-time PCR analyses were carried out to detect the expression of VEGF, MMP9 and TGF-β in tumor tissues. We found that the expression of all these genes in co-injected groups were higher than that in HGC-27 cells alone group (Figure 5D). The results of real-time PCR analyses showed that the mRNA levels of Oct4, Sox2 and Sall4 were the highest in activated MSCs co-injected group (Figure 5E). In summary, these data indicate that macrophages-activated MSCs prompt gastric cancer growth *in vivo*.

### Primary Monocytes Activated MSCs to Prompt Gastric Cancer Cell Proliferation and Migration Through NF-κB

**Figure 6:**
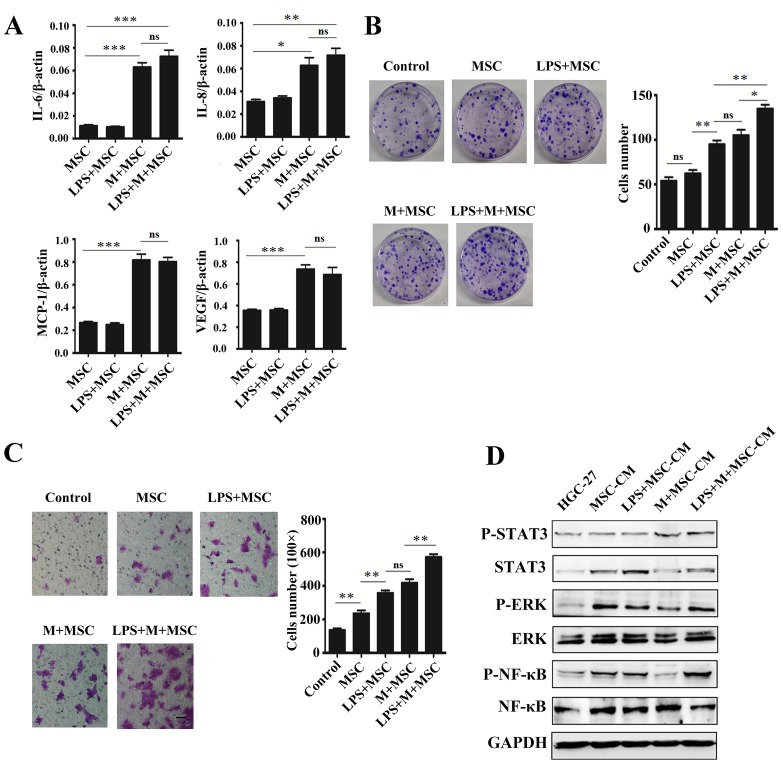
Primary monocytes derived macrophages activated MSCs to enhance gastric cancer cell proliferation and migration through NF-κB. (A) Real-time PCR analyses of IL-6, IL-8, MCP-1, and VEGF mRNA expression in MSCs. ***P<0.001, **P<0.01, *P<0.05. (B) Representative images of cell colonies and histogram of colony number. Data were listed as mean±SD of three wells. **P<0.01, *P<0.05. (C) Representative images of transwell migration assay and histogram of the number of migrated HGC-27 cells. Magnification, ×100; scale bar = 50 µm. **P<0.01. (D) Western blot assays for p-STAT3, STAT3, p-ERK, ERK, p-NF-κB, and NF-κB protein levels in HGC-27 cells.

To further confirm the activation of MSCs by macrophages to prompt gastric cancer cell proliferation and migration, we isolated monocytes from human peripheral blood and collected the supernatant from monocytes differentiated macrophages. After incubation with the supernatant from monocytes differentiated macrophages, MSCs were harvested and the total RNA was extracted for real-time PCR analyses. As shown in Figure 6B, treatment with the supernatant from monocytes differentiated macrophages up-regulated the mRNA levels of IL-6, IL-8, MCP-1 and VEGF in MSCs. We then incubated gastric cancer cells with the supernatant from the activated MSCs. The results of cell colony formation and transwell migration assays showed that the supernatant from the activated MSCs enhance the proliferation and migration of HGC-27 cells (Figures 6C and 6D). We further demonstrated that incubation with supernatants from the activated MSCs increased the expression of p-STAT3, p-ERK and p-NF-κB in HGC-27 cells (Figure 6E). Taken together, these data suggest that in consistent with the THP-1 cells, human primary macrophages also activated MSCs to prompt gastric cancer cell proliferation and migration through NF-κB.

## Discussion

During the past decades, the link of inflammation to cancer has attracted a lot of attention [5], [6]. Long-term exposure of epithelial cells to inflammatory microenvironment leads to malignant transformation [9], [19]. The stromal cells have been shown to be critically involved in the progression from inflammation to cancer by modulating the inflammatory microenvironment [7], [8]. Macrophages and MSCs are two major components of tumor stroma and participate in regulating the extent of inflammatory reaction during carcinogenesis [16]. However, the interplay between macrophages and MSCs in gastric cancer has not been well characterized.

Gastric cancer is evolved from long-term chronic gastritis and is a classic model of inflammation-related cancer. We have previously isolated resident MSCs from human gastric cancer tissues and demonstrated that gastric cancer tissue derived MSCs secreted lots of inflammatory cytokines and promoted gastric cancer progression. During the process of inflammation, monocytes/macrophages could be recruited to endow MSCs with tumor-promoting features [16]. In addition, *Helicobacter pylori* induce chronic inflammation in the gastric epithelial cells through the release of LPS existing in its cell walls. In this study, we pre-treated MSCs with macrophages in the presence of LPS to mimic gastric cancer microenvironment and to determine whether macrophages-activated MSCs contribute to gastric cancer progression. We demonstrated that MSCs incubated with macrophages and LPS secreted higher levels of inflammatory cytokines. A recent study reported that continuous stimulation with macrophage conditioned medium induced MSCs to acquire a pro-inflammatory phenotype [17]. In consistent with their study, we found that short time incubation with macrophages also activated MSCs to produce higher level of inflammatory cytokines.

Epithelial-mesenchymal-transition (EMT) is a significant process during cancer metastasis [20]. Over the years, reprogramming properties related to EMT have been attributed to enhance cellular plasticity in cancer cells. Cancer stem cell has been introduced and shown to contribute to tumorigenesis and tumor metastasis [21]–[24]. Recent studies suggest that a subpopulation of stem-like and mesenchymal-like cells displayed a greater tumorigenicity in gastric epithelial cells *in vitro and in vivo* [25]. In this study, we found that activated MSCs, which were co-cultured with macrophages in the presence of LPS, could remarkably enhance the migration of gastric epithelial cells as well as gastric cancer cells. Furthermore, we demonstrated that supernatants from the activated MSCs induced EMT in gastric epithelial and gastric cancer cells. To further explain these results, we detected the expression of stemness genes in gastric cancer cells. We found that the expression of Oct4 and Sox2, two major stemness-related genes, was obviously increased by the activated MSCs. Angiogenesis is critical for tumor metastasis and the expression of VEGF is closely related to gastric cancer prognosis [26], [27]. We found that VEGF expression was significantly increased after treatment with the supernatants from the activated MSCs in gastric cancer cells both *in vitro* and *in vivo*. Based on these findings, we proposed that macrophages-activated MSCs secreted more inflammatory cytokines and promoted the proliferation and migration of gastric cancer cells by the induction of EMT and cell stemness.

Several studies have indicated that MSCs promote gastric cancer growth through different pathways [28], [29] and a recent study has demonstrated that VEGF expression is closely related to NF-κB in gastric cancer [30]. Moreover, TGF-β plays an important role in controlling cell proliferation, apoptosis, differentiation, migration, and extracellular matrix development [31], [32]. Recent researches have revealed that TGF-β activates NF-κB and plays important roles in the progress of synergistic activation of various pathways [33], [34]. TGF-β-mediated induction of matrix metalloproteinase 9 (MMP9) has been reported to be regulated by NF-κB and JNK pathways [35]. TNF-stimulated MMP9 production also activates NF-κB and ERK signaling pathways [36]. Additionally, the progression of gastric epithelial cells to gastric cancer cells has been shown to be regulated by cytokine expression and NF-κB signaling pathway [37]. Another report suggests that inhibiting both proliferation and angiogenesis with drug intervention in gastric cancer is related to the suppression of NF-κB [38]. In this study, we showed that macrophages-activated MSCs up-regulated the phosphorylation of NF-κB and the expression of VEGF, TGF-β, and MMP9 in gastric cells. The simultaneous increases in STAT3 and ERK phosphorylation may result from the activation of NF-κB because NF-κB has been previously shown to regulate STAT3 and ERK signaling pathways [39], [40].

In conclusion, we demonstrated in this study that MSCs activated by macrophages acquired pro-inflammatory phenotype and promoted gastric epithelial cell and cancer cell proliferation and migration through NF-κB activation. Our findings provide novel evidence for the modulation of gastric epithelial cells and cancer cells by MSCs under inflammatory environment and further insight to the roles of stromal cells in the progression from chronic inflammation to cancer.

## Supporting Information

Figure S1 **Co-culture of MSCs with THP-1 cells.** Representative images of THP-1 cells and MSCs in a direct co-culture system before (left) and after (right) PBS washing. Magnification, ×100, scale bar = 50 µm.
(TIF)

Figure S2 **Macrophages-activated MCSs induced the activation of NF-κB in SGC-7901 cells.** SGC7901 cells were treated with the supernatants from macrophages-activated MSCs and the expression of p-STAT3, STAT3, p-ERK, ERK, p-NF-κB, and NF-κB proteins in SGC-7901 cells were detected by using Western blot.
(TIF)

Table S1 **List of primer sequences.**
(DOC)
