# Supplementary figures and images for: Activation of Mesenchymal Stem Cells by Macrophages Prompts Human Gastric Cancer Growth through NF-κB Pathway
Source: PLoS One. 2014 May 13;9(5):e97569. doi: 10.1371/journal.pone.0097569 (PMC4019592; doi:10.1371/journal.pone.0097569)

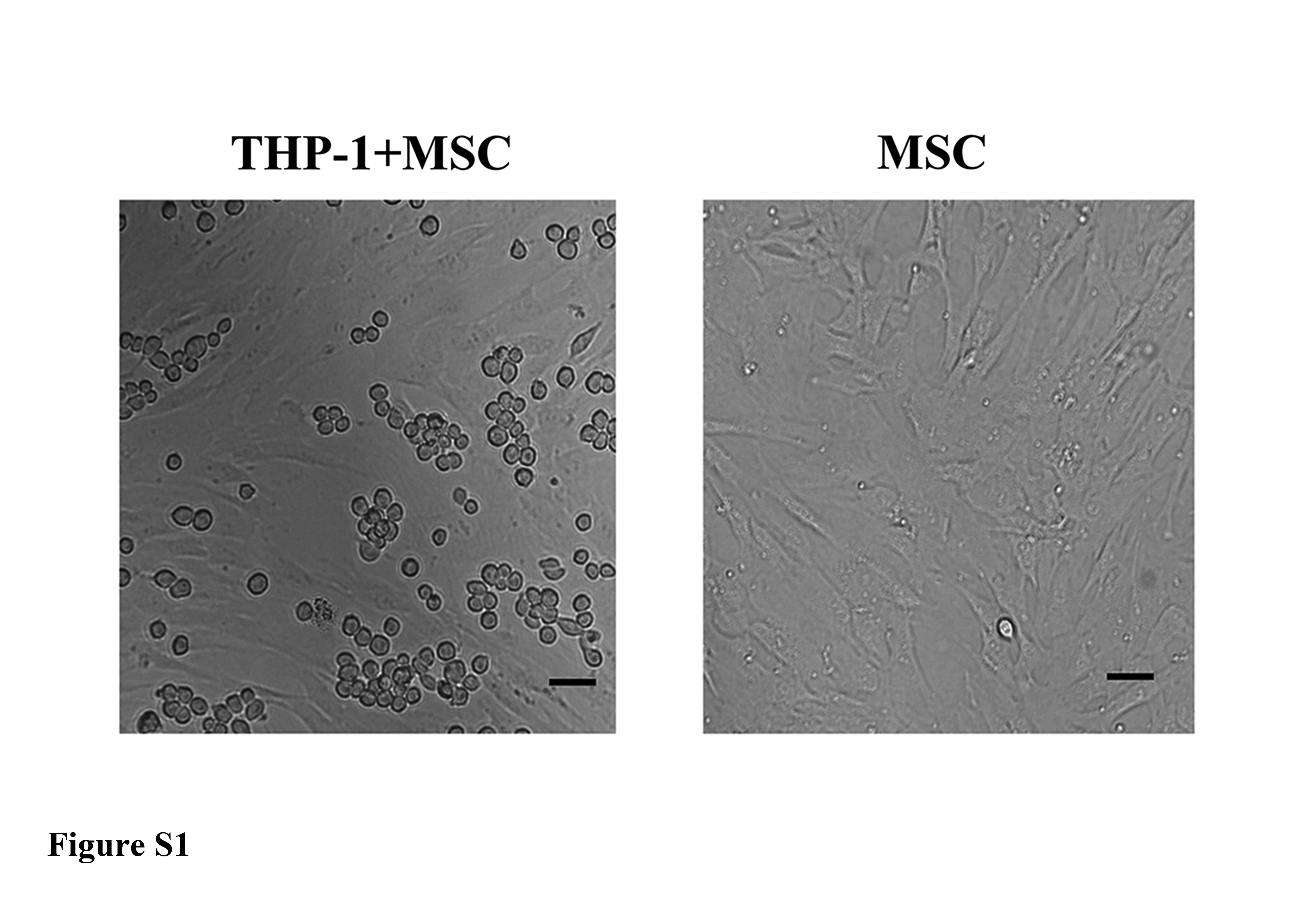

Supplement: Figure S1 — Co-culture of MSCs with THP-1 cells. Representative images of THP-1 cells and MSCs in a direct co-culture system before (left) and after (right) PBS washing. Magnification, ×100, scale bar = 50 µm. (TIF) [file pone.0097569.s001.tif]

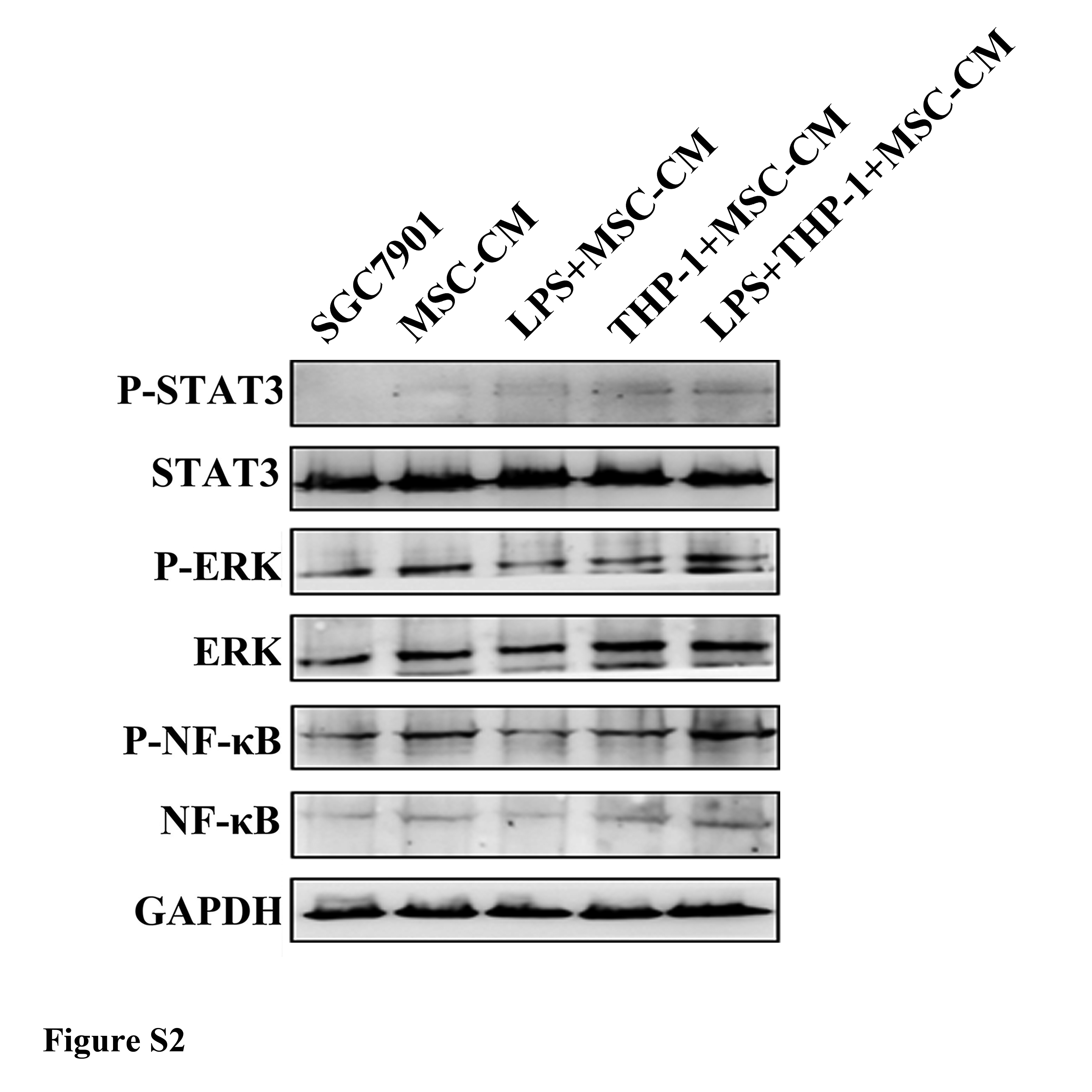

Supplement: Figure S2 — Macrophages-activated MCSs induced the activation of NF-κB in SGC-7901 cells. SGC7901 cells were treated with the supernatants from macrophages-activated MSCs and the expression of p-STAT3, STAT3, p-ERK, ERK, p-NF-κB, and NF-κB proteins in SGC-7901 cells were detected by using Western blot. (TIF) [file pone.0097569.s002.tif]
