# Supplementary material for: Activation of Mesenchymal Stem Cells by Macrophages Prompts Human Gastric Cancer Growth through NF-κB Pathway
Source: PLoS One. 2014 May 13;9(5):e97569. doi: 10.1371/journal.pone.0097569 (PMC4019592; doi:10.1371/journal.pone.0097569)
Supplement: Table S1 — List of primer sequences. (DOC) [file pone.0097569.s003.doc]

**Table S1. List of primer sequences**

| Genes name | Forward primer | Tm(^o^C) | Length(bp) |
| --- | --- | --- | --- |
|  | Reverse primer |  |  |
| IL-6 | TACATCCTCGACGGCATCTC AGCTCTGGCTTGTTCCTCAC | 61 | 252 |
| IL-8 | GCTCTGTGTGAAGGTGCAGTTT TTCTGTGTTGGCGCAGTGT | 62 | 144 |
| TNF-α | CCGAGTGACAAGCCTGTAGC AGGAGGTTGACCTTGGTCTG | 57 | 493 |
| MCP-1 | GAACCGAGAGGCTGAGACTA GCCTCTGCACTGAGATCTTC | 59 | 259 |
| VEGF | CCTTGCTGCTCTACCTCCAC ATCTGCATGGTGATGTTGGA | 58 | 280 |
| TGF-β | CACACTGCAAGTGGACATC GCAGAAGTTGGCATGGTAG | 57 | 277 |
| MMP9 | ACGTCTTCCAGTACCGAGAG GGCACTGCAGGATGTCATAG | 60 | 126 |
| Oct4 | TTGAGGCTCTGCAGCTTAG GCCGGTTACAGAACCACAC | 60 | 285 |
| Sox2 | ACACCAATCCCATCCACACT GCAAACTTCCTGCAAAGCTC | 60 | 224 |
| Sall4 | TCGATGGCCAACTTCCTTC GAGCGGACTCACACTGGAGA | 62 | 142 |
| β-actin | CACGAAACTACCTTCAACTCC CATACTCCTGCTTGCTGATC | 56 | 265 |
